# Low levels of genetic differentiation and structure in red fox populations in Eastern Canada and implications for Arctic fox rabies propagation potential

**DOI:** 10.1371/journal.pone.0286784

**Published:** 2023-06-06

**Authors:** Benoit Talbot, Thaneah J. Alanazi, Vicky Albert, Émilie Bordeleau, Émilie Bouchard, Patrick A. Leighton, H. Dawn Marshall, Daphné Rondeau-Geoffrion, Audrey Simon, Ariane Massé

**Affiliations:** 1 School of Epidemiology and Public Health, University of Ottawa, Ottawa, ON, Canada; 2 Research Group on Epidemiology of Zoonoses and Public Health (GREZOSP), Faculty of Veterinary Medicine, Université de Montréal, Saint-Hyacinthe, QC, Canada; 3 Department of Biology, Memorial University of Newfoundland, St. John’s, NL, Canada; 4 Ministère des Forêts, de la Faune et des Parcs, Québec, QC, Canada; 5 Department of Microbiology, Western College of Veterinary Medicine, University of Saskatchewan, Saskatoon, SK, Canada; University of Illinois at Urbana-Champaign, UNITED STATES

## Abstract

Rabies is a lethal zoonosis present in most parts of the world which can be transmitted to humans through the bite from an infected mammalian reservoir host. The Arctic rabies virus variant (ARVV) persists mainly in populations of Arctic foxes (*Vulpes lagopus*), and to a lesser extent in red fox populations (*Vulpes vulpes*). Red foxes are thought to be responsible for sporadic southward movement waves of the ARVV outside the enzootic area of northern Canada. In this study, we wanted to investigate whether red foxes displayed notable levels of genetic structure across the Quebec-Labrador Peninsula, which includes portions of the provinces of Quebec and Newfoundland-Labrador in Canada, and is a region with a history of southward ARVV movement waves. We combined two datasets that were collected and genotyped using different protocols, totalling 675 red fox individuals across the whole region and genotyped across 13 microsatellite markers. We found two genetic clusters across the region, reflecting a latitudinal gradient, and characterized by low genetic differentiation. We also observed weak but significant isolation by distance, which seems to be marginally more important for females than for males. These findings suggest a general lack of resistance to movement in red fox populations across the Quebec-Labrador Peninsula, regardless of sex. Implications of these findings include additional support for the hypothesis of long-distance southward ARVV propagation through its red fox reservoir host.

## Introduction

Rabies is a lethal zoonosis in mammals, including humans, present in most parts of the world [1, 2]. The disease is transmitted from an individual to another through physical contact, mainly through broken skin or through broken lining of the mouth and nose [2]. The majority of rabid individuals exhibit a hyperexcitable behavior, while the remainder typically displays a flaccid paralytic form of the disease, and both forms are followed by encephalopathy and death [1]. Emergence of rabies has occurred in a number of mammal species, which lead to genetic divergence and new enzootic transmission cycles in the new reservoir species [3–5]. Raccoons, foxes, skunks and coyotes have been identified as terrestrial mammal hosts of various strains of the virus in North America in the recent decades [6].

Rabies has been documented in the Canadian Arctic, Greenland and Svalbard for a long time, known to be transmitted by Arctic foxes (*Vulpes lagopus*) to dogs and people [7]. The Arctic rabies virus variant (ARVV) has been enzootic in northern Canada over the last 60 years [8], and is thought to be persisting mainly in populations of Arctic foxes, despite its low density across its distribution range [9–11]. It has been isolated in a number of other mammalian species throughout the Arctic, but also in more southerly areas [11, 12]. The red fox (*Vulpes vulpes*) is thought to be a competent reservoir species for the ARVV, although the virus strain does not seem to persist to the same extent in red fox as it does in Arctic fox populations [12]. Several past rabies outbreaks in southern Canada, namely in Ontario, Quebec and Newfoundland [13–15] traced their origin to the ARVV. Specifically, southward movement of the ARVV, in multiple waves mainly through transmission among Arctic and red foxes, has been documented since 1956 in the Quebec-Labrador Peninsula, which includes northern Quebec and Labrador [16–18]. Furthermore, as average temperatures increase, expected through climate change, we predict a decrease in reported rabies cases in Arctic foxes [10], followed by a potential spread of the ARVV to the south via red foxes [19].

Understanding how pathogens spread over the environment is of crucial importance for implementing disease management efforts during outbreaks of zoonoses [20, 21]. We expect that propagation of pathogens transmitted through bite from infected hosts will be tightly linked to dispersal of their hosts. Therefore, management efforts will need information about movements of hosts of a disease in order to inform practical measures to effectively control its spatial propagation, when possible. For example, during an epidemic of the raccoon rabies virus variant in early 2000s in southern Ontario and Quebec, major rivers were found to have an impact on the dispersal of the main reservoir species of the raccoon rabies virus variant, the raccoon (*Procyon lotor*) and the striped skunk (*Mephitis mephitis*) [22–24]. Therefore, vaccination efforts by government authorities to contain the virus variant were deployed mainly around these landscape features, to increase odds of containing the propagation waves along these landscape elements.

Investigations of the population genetics of Arctic and red foxes suggest a low degree of genetic differentiation in both species, and almost no effect of geographic distance on gene flow [25–27]. Several landscape components, including tundra versus boreal landscape boundaries [25], prey community structure [26] and forest and water patch distributions [27] have been identified as potentially responsible for the cryptic levels of genetic structure observed in both species. In this study, we wanted to investigate whether red foxes displayed notable levels of genetic structure across the Quebec-Labrador Peninsula and southern regions of Quebec. Based on prior findings in other regions, we expected low levels of genetic differentiation and spatial genetic structure in the species across the Quebec-Labrador Peninsula. Given this region has been implicated in a number of southerly rabies propagation waves into highly populated areas of southeastern Canada, results from such an investigation could prove useful to gain insights into the processes driving rabies propagation in this region, especially in the midst of climate change.

## Materials and methods

### Data collection

**Fig 1:**
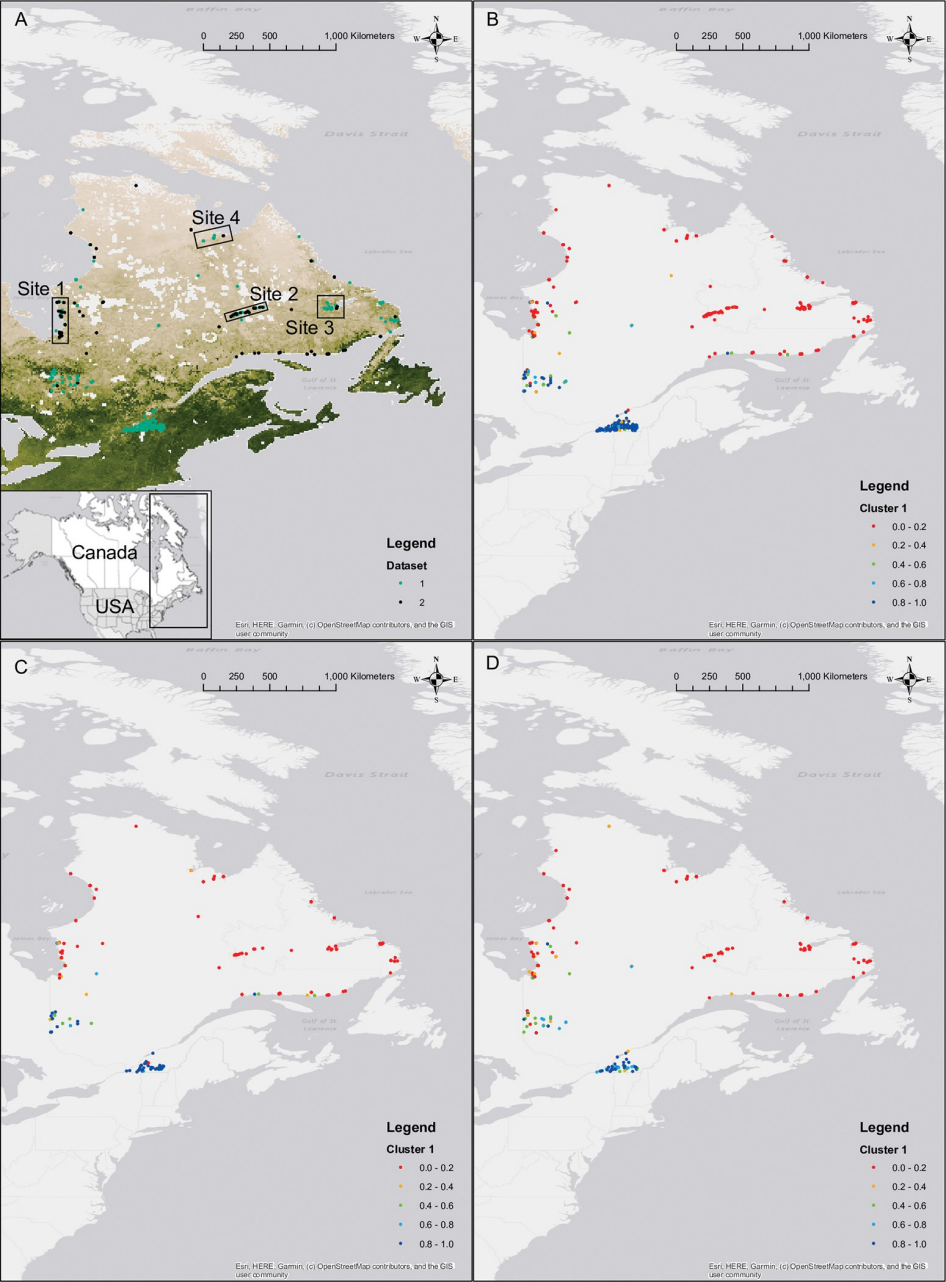
Geographical map of red fox (*Vulpes vulpes*) individuals collected across the Quebec-Labrador Peninsula in Canada from 2012 to 2018. Individuals from the two datasets (A; n = 675) used for this study are represented as in Legend, and normalized difference vegetation index [29, 30] is represented in a gradient from deep green (extensive forest) to white (barren rock). All individuals (B; n = 675), female individuals (C; n = 250) and male individuals (D; n = 321) are represented based on their probability of membership to Cluster 1, based on results from a Structure [31] approach using data from 13 microsatellite markers, which identified two genetic clusters in all three analyses. Map was created using ArcGIS 10.5 (ESRI, Redlands, CA, USA).

For this study, we used two sets of red fox samples collected as part of two different projects, totalling 675 individuals. The first dataset includes 365 individuals collected from two sources, from 2012 to 2017 across the Quebec-Labrador Peninsula and southern regions of Quebec (Fig 1A; S1 Table). A portion of these samples were originally collected as part of provincial and federal rabies surveillance operations across the provinces of Quebec and Newfoundland-Labrador in Canada by the Ministère des Forêts, de la Faune et des Parcs Quebec government agency, the Canadian Food Inspection Agency and the Government of Newfoundland-Labrador. The rest of the samples were collected through the ArcticNet Networks of Centres of Excellence (Université Laval, Québec, QC, Canada). These samples were donated to be used as part of an MSc thesis at the Memorial University of Newfoundland [28]. The second dataset includes 310 individuals collected as part of a PhD thesis at the University of Saskatchewan, using the help of local trappers and municipal authorities, from 2016 to 2018 across the Quebec-Labrador Peninsula and southern regions of Quebec (Fig 1A; S1 Table). In both datasets, tissue samples were collected mostly from trapped individuals, and also opportunistically from dead animals. No permit was required for collection of all specimens used in this study, because they were all obtained either through collection of carcasses, or collection of trapped or hunted live animals. The Ministère des Forêts, de la Faune et des Parcs Quebec government agency’s legislation allows collection of such specimens for wildlife surveillance or wildlife management purposes.

### Genetic analyses

For both datasets, DNA was extracted from tissue samples using the DNeasy Blood & Tissue Kit (QIAGEN, Germantown, Maryland, United States), according to the manufacturer’s instructions. Across the two datasets, we used four thermocyclers: Mastercycler Pro S, Mastercycler X50a (Eppendorf, Hamburg, Germany), 2720 Thermal Cycler and SimplyAmp (Applied Biosystems; ABI, Foster City, California, United States) to execute the Polymerase Chain Reaction (PCR) amplification of 15 microsatellite markers developed for a range of canid species, including domestic dogs, Arctic fox and red fox. Specific PCR program and recipe used to genotype both datasets are detailed by Alanazi, 2020 [28]. For the first dataset, we sized PCR products on a Kodak Gel Logic 200 Imaging System and microsatellite genotypes were called using the PeakScanner software 1.0 (ABI, Foster City, California, United States). For the second dataset, we sized PCR products on a 3130 Genetic Analyzer (ABI, Foster City, California, United States) and microsatellite genotypes were called using the GeneMapper software 4.0.2 (ABI, Foster City, California, United States). Standardization of allele calls was performed using a subset of 30 samples from Dataset 1 before proceeding to the genotyping of samples from Dataset 2.

### Suitability of microsatellite markers and datasets

We selected four locations (Sites 1–4; Fig 1A) with a high spatial density of red fox samples taken from the two datasets (n = 252). We used individuals from these locations to test suitability of our microsatellite markers for population genetic analyses and to test whether differences in genotyping equipment and procedure used lead to differences attributable to the dataset of origin, which could preclude using a combined dataset for population genetic analyses.

First, we tested all microsatellite markers for Hardy-Weinberg and linkage disequilibrium, using GENEPOP 4.7.5 [32], on samples from Sites 1–4 separated according to each sample’s collection site. For each type of test, we corrected for multiple tests using Bonferroni correction, with a threshold α of 0.05. We also computed average number of alleles, average observed and expected heterozygosities, average inbreeding coefficient G_IS_, and average Hedrick’s global population differentiation index G’_ST_, across sites, for each microsatellite marker, using samples from Sites 1–4. To determine whether our selection of sites was representative of the whole dataset, we calculated average number of alleles, and average observed and expected heterozygosities, for the combined dataset of 675 individuals, with no separation according to collection site.

Secondly, we tested whether inclusion of samples from the two datasets lead to differences attributable to the dataset of origin, using two approaches on data from Sites 1–4. We tested each site for Hardy-Weinberg and linkage disequilibrium, using GENEPOP 4.7.5 [32]. We also used the Bayesian clustering algorithm implemented in the Structure 2.3.4 software [31] on samples from each site separately. We expected no Hardy-Weinberg and linkage disequilibrium and a single genetic cluster in each site. Lastly, using GenoDive 3.0.1, we calculated Hedrick’s global population differentiation index G’_ST_ and genetic variation using analysis of molecular variance, between the two datasets, for Sites 1–4.

### Genetic differentiation and spatial genetic structure

Using the combined dataset, we looked for evidence of genetic differentiation in the study area using two clustering algorithms. We ran these analyses three times: for all individuals, for females only and for males only. In case of a number of genetic clusters (K) > 1, we assigned the most likely cluster to each individual, and we calculated Hedrick’s G’_ST_ and conducted an analysis of molecular variance to determine the extent of genetic differentiation among genetic clusters, using GenoDive 3.0.1.

First, we used the Bayesian clustering algorithm implemented in the Structure 2.3.4 software [31]. We did not use site of collection as prior population information. We computed probability of data to estimate the number of genetic clusters (*K*), using 100,000 iterations, and a burn-in period of 10,000 iterations. We executed 10 runs for each *K* value between 1 and 10. We used the admixture model, individual alpha for each population, allele frequencies correlated among populations and inferred a separate lambda for each population. We kept all other parameters at default value. We used the CLUMPAK software [33], in which is implemented the Evanno et al.’s [34] method, to determine the most probable *K*. We executed the analysis a second time, using the most probable *K* determined in the prior analysis, to calculate membership probability of each individual to each identified genetic cluster.

Secondly, we ran an analysis using the ‘adegenet’ package in R 4.2.1 [35], which implements a spatial principal components analysis-based clustering algorithm. We ran the ‘spca’ function using the genetic and spatial data associated with each sample, and selected the Delaunay triangulation connection network. We retained the number of eigenvalues showing an extremely high or low value compared to the rest of the distribution. We used the ‘global.rtest’ function, using 999 permutation steps, to determine whether the global and/or local patterns of genetic structure was significant. We plotted the distribution of lag vectors to determine the number of genetic clusters visually. We used ‘Moran.I’ function from the ‘ape’ R package [36] in R. 4.2.1 to compute Moran’s I autocorrelation coefficient to investigate the presence of spatial autocorrelation in the data.

We also looked for evidence of isolation by distance across the study area, using a matrix-based linear multiple regression approach. We measured the effect of geographic distance on genetic distance. We calculated Smouse and Peakall’s [37] individual genetic distance for each pair of individuals, using GenoDive 3.0.1. We calculated geographic distance (in km) for each pair of individuals, corrected for sphericity of the earth, using the ‘rdist.earth’ function from the ‘fields’ package [38] in R 4.2.1 (R Development Core Team, Vienna, Austria). We then fit pairwise genetic distance to geographic distance using a multiple regression on distance matrices, using the ‘MRM’ function from the ‘ecodist’ package [39] in R 4.2.1, which uses a Mantel test derived linear regression model. We assessed significance through a permutation procedure (999 replicates) that takes into account non-independence of data points in distance matrices [40, 41]. We ran the analysis three times: for all individuals, for females only and for males only. For each analysis, we used a jackknife procedure to compute a standard error, which we used to compute a 95% confidence interval of the slope estimate. These confidence intervals can be used to compare slopes across analyses, and determine whether genetic distance is affected differently by geographic distance for each sex.

## Results

### Suitability of microsatellite markers and datasets

Using data from Sites 1–4 (n = 252; Fig 1A), we found significant deviations of Hardy-Weinberg equilibrium at two out of 15 markers, namely at markers CPH3 and CXX173 (Table 1), at a threshold α = 0.05 after Bonferroni correction. After removing these two markers, no significant deviation of Hardy-Weinberg equilibrium was observed. Using data from the remaining 13 markers, we found one significant case of linkage disequilibrium across all pairs of markers, at a threshold α = 0.05 after Bonferroni correction. This unique deviation pertains to markers AHT121 and CXX377 from Site 4. Since this deviation is not uniform across sites, we decided to retain the two markers for our analyses. Average number of alleles varied between 5 and 13 across markers. Average observed and expected heterozygosities were mostly high, and average inbreeding coefficients and average global differentiation indexes were mostly low (Table 1).

**Table 1:** Average number of alleles (N_A_), average observed and expected herozygosities (H_O_ and H_S_), average inbreeding coefficient (G_IS_), average Hedrick’s global population differentiation index (G’_ST_ Hed) and Hardy-Weinberg Equilibrium test *P* value (HWE *P*), calculated across sampling sites, for 15 microsatellite markers genotyped for 252 red fox (*Vulpes vulpes*) individuals from Sites 1–4, as in Fig 1A, collected from 2012 to 2018 in the Quebec-Labrador Peninsula in Canada.

Locus	N_A_	H_O_	H_S_	G_IS_	G’_ST_ Hed	HWE *P*
AHTh171	5	0.402	0.400	0.000	0.014	0.972
Co4_140	9	0.650	0.632	0.000	0.010	0.191
REN105L03	8	0.846	0.812	0.000	0.024	0.486
AHT121	13	0.840	0.841	0.001	0.000	0.859
CPH3	8	0.508	0.550	0.077	0.003	< 0.001*
REN247M	10	0.654	0.724	0.096	0.000	0.112
Co1_424	5	0.610	0.598	0.000	0.008	0.157
CPH15	8	0.728	0.751	0.032	0.097	0.508
CPH9	6	0.554	0.592	0.064	0.000	0.241
CXX109	4	0.601	0.612	0.018	0.009	0.686
CXX250	8	0.682	0.706	0.034	0.000	0.271
CXX172	6	0.747	0.744	0.000	0.077	0.424
CXX173	5	0.207	0.242	0.144	0.000	0.002*
CXX20	9	0.770	0.748	0.000	0.002	0.831
CXX377	9	0.682	0.740	0.079	0.031	0.594
Overall	8	0.632	0.646	0.022	0.014	0.007*

*Significant at ⍺ = 0.05 after Bonferroni correction

Using data from 13 markers, we found no significant deviation of Hardy-Weinberg equilibrium in Sites 1–4, at a threshold α = 0.05 after Bonferroni correction (Table 2). We observed highest probability of a single genetic cluster in Sites 1–4 (Table 2; S1 Appendix). Genetic differentiation index and genetic variation between the two datasets was extremely low for Site 1–4 (Table 2). Therefore, we concluded that combining the two datasets was appropriate for the remainder of our analyses.

**Table 2:** Hardy-Weinberg Equilibrium test P value (HWE *P*), most likely number of genetic clusters (*K*) identified by Bayesian clustering algorithm, and genetic differentiation index (G’_ST_ Hed) and genetic variation (%) among datasets, using data from 13 microsatellite markers genotyped for 252 red fox (*Vulpes vulpes*) individuals from Sites 1, 2, 3 and 4, as in Fig 1A, collected from 2012 to 2018 in the Quebec-Labrador Peninsula in Canada.

Site	Region	Sample size			HWE *P*	*K*	G’_ST_ Hed	%
		*Dataset 1*	Dataset 2	Total				
Site 1	James Bay coast	20	55	75	0.141	1	0.01	0.7
Site 2	Central Labrador	33	23	56	0.882	1	0.00	0.0
Site 3	Goose Bay town	43	24	67	0.385	1	0.00	0.2
Site 4	Kuujjuaq town	23	31	54	0.718	1	0.02	0.0

Across the combined dataset, average number of alleles was 8, observed heterozygosity was 0.658, and expected heterozygosity was 0.700. All these values are similar to those calculated for Sites 1–4 (Table 1).

### Genetic differentiation and spatial genetic structure

Using the combined dataset of 675 red fox individuals, our genetic clustering analysis using both approaches (i.e. Bayesian clustering algorithm and spatial principal components analysis-based clustering algorithm) showed highest probability of a total of two clusters, when analyzing all individuals, females only and males only (S1 Appendix). There was concordance in genetic cluster assignment across samples between approaches (Fig 1; S1 Appendix). Interestingly, membership to the two genetic clusters was distributed along a latitudinal gradient across the study area in all three analyses (Fig 1B–1D). Genetic cluster membership distribution seemed to spatially match with the distribution of normalized difference vegetation index [29, 30], which is shown as a gradient in Fig 1A. Indeed, most individuals with strongest membership probability with Cluster 1 are located in areas with a high normalized difference vegetation index in the south of the study area, and most individuals with strongest membership probability with Cluster 2 are located in areas with a comparatively low normalized difference vegetation index in a large area in the north of the study area.

Hedrick’s G’_ST_ value between the two genetic clusters was 0.225, 0.248 and 0.272, for males only, all individuals, and females only, respectively. Analysis of molecular variance showed 3.1%, 3.9% and 5.5% of genetic variation was among genetic clusters, for males only, all individuals, and females only, respectively. These results suggest low but notable levels genetic differentiation between the identified two genetic clusters for all three analyses.

**Fig 2:**
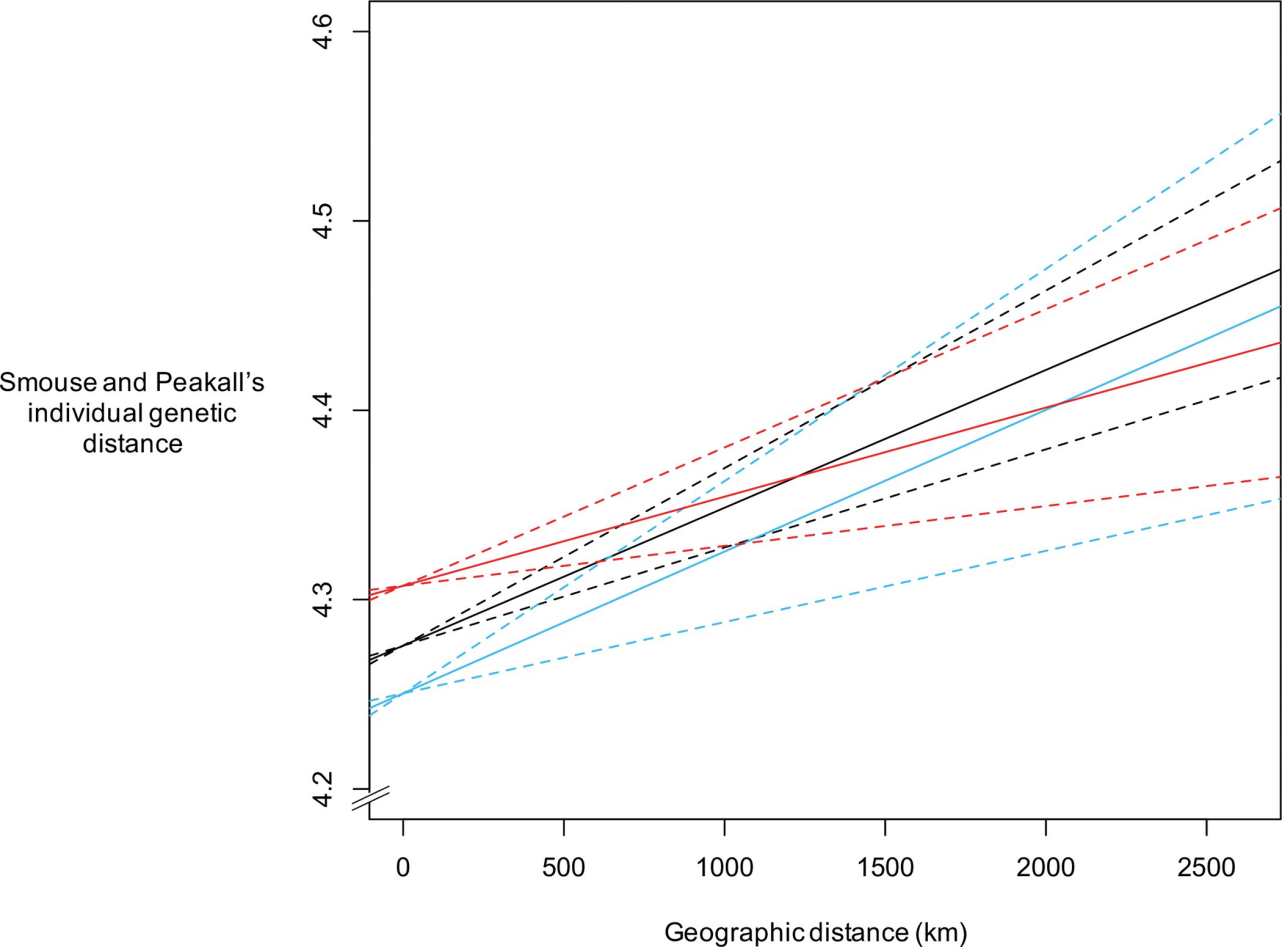
Graph (slope in solid line; 95% confidence intervals in dashed lines) of three analyses (all individuals in black; females only in blue; males only in red) of multiple regression on distance matrices on the effect of geographic distances (km) on Smouse and Peakall’s individual genetic distances, using data from 13 microsatellite markers genotyped for 675 red fox (*Vulpes vulpes*) individuals collected across the Quebec-Labrador Peninsula in Canada, as in Fig 1A, from 2012 to 2018.

Our multiple regression on distance matrix analyses revealed a significant effect of geographic distance on genetic distance, for all individuals, for females only and for males only (Table 3). The slope estimates were small in all three analyses (Fig 2), suggesting weak but significant isolation by distance. The slope estimate was slightly larger for females than for males, but overlapping 95% confidence intervals suggest a marginal difference (Table 3; Fig 2). These results suggest strong gene flow across the study area, regardless of sex. Moran’s I autocorrelation coefficient was high and significant for all individuals, females only and males only (Table 4), suggesting presence of spatial autocorrelation in our study area. These results support results from the multiple regression on distance matrix analyses.

**Table 3:** Slope (95% confidence interval), *P* and R^2^ values for three analyses of multiple regression on distance matrices on the effect of geographic distances (km) on Smouse and Peakall’s individual genetic distances, using data from 13 microsatellite markers genotyped for 675 red fox (*Vulpes vulpes*) individuals collected across the Quebec-Labrador Peninsula in Canada, as in Fig 1A, from 2012 to 2018.

Analysis	Slope (95% CI)	*P*	R^2^
All individuals	0.000073 (0.000011. 0.000052)	< 0.001	0.011
Females only	0.000075 (0.000019. 0.000038)	< 0.001	0.012
Males only	0.000047 (0.000013. 0.000021)	< 0.001	0.005

**Table 4:** Observed and expected Moran’s I autocorrelation coefficient, standard deviation (SD), and *P*, using data from 13 microsatellite markers genotyped for 675 red fox (*Vulpes vulpes*) individuals collected across the Quebec-Labrador Peninsula in Canada, as in Fig 1A, from 2012 to 2018.

Analysis	Observed	Expected	SD	*P*
All individuals	0.703	-0.001	0.007	< 0.001
Females only	0.675	-0.004	0.017	< 0.001
Males only	0.658	-0.003	0.013	< 0.001

## Discussion

Our study investigated the genetic structure of red foxes across the Quebec-Labrador Peninsula and southern regions of Quebec. Based on previous reports, we expected weak genetic differentiation and weak spatial genetic structure across the study area, despite its large extent [25, 42–44]. Our findings support our predictions, where small but significant genetic differentiation was observed between two clusters distributed in a latitudinal gradient across the Quebec-Labrador Peninsula and southern regions of Quebec. One genetic cluster is mostly distributed across the extreme south of the study area, whereas the other mostly spans the entire rest of the study area. We also observed a weak but significant effect of geographic distance on genetic distance, suggesting large geographic distances only have limited effect on gene flow in red foxes across the landscape. Similar results were found in red foxes in Alaska, where weak isolation by distance was observed, and genetic cluster boundaries seem to match tundra versus boreal landscape boundaries [25]. Dispersal distance in mammals is associated mainly with the size of the species, its home range and range distribution [45]. In carnivore species such as foxes, seasonal shifts in resource availability lead to frequent long-distance dispersal behaviors [46].

Our findings of a marginally lower slope estimate of isolation by distance for males, and slightly lower genetic differentiation among genetic clusters for males, suggest the presence of slightly male-biased dispersal. Altogether, the huge geographic distances surveyed in our study did not seem to affect either sex to a large extent. In most mammal species, we observe female philopatry and male-biased dispersal [47], although female-biased dispersal is also observed in many species [48]. In red foxes, evidence from the current literature support both male-biased dispersal [49] and female-biased dispersal [50].

Our results also highlight the larger long-distance movement potential in red foxes compared to two other mesocarnivorous rabies reservoir species such as striped skunk and raccoon, which showed a larger slope estimate of isolation by distance across a much smaller but partially spatially overlapping study region [22, 23, 51]. On the other hand, striped skunks and raccoons usually display much higher population density where they occur. Although greater population density may largely increase local rabies transmission potential, long-distance movements may contribute to spatial rabies propagation potential. Our results therefore suggest risks associated with rabies spread could vary depending on reservoir species involved at any given point in time. Careful considerations of these concepts should therefore be considered when designing rabies surveillance and control interventions.

One major limitation of our study is low sampling in the interior of the study region. Reasons for this sampling bias include lack of human settlements away from the coast in northern Canada, and lack of transportation options to reach the interior areas. However, the typical long-distance dispersal behavior seen in red foxes in general and our findings of a single genetic cluster across most of the northern portion of the Quebec-Labrador Peninsula give us confidence that we were able to capture most of the genetic variation present in this area. Another limitation is our use of two datasets collected for different studies and genotyped using different methods. However, our site-specific analysis was unable to parse the two datasets into separate populations, across four sites. Altogether, we are confident that using two datasets for our study did not reduce the representativity of our results. Finally, almost all red fox samples used in this study either tested negative for rabies, or were not tested, and rabid animals may display a hyperexcitable or a paralytic behavior, and may therefore hypothetically disperse over larger or smaller distances than non-rabid individuals, respectively, during these disease stages. However, rabies symptoms only appear in the late infection stages several days before death occurs. Therefore, infection status is unlikely to have a significant effect on genetic structure, as hypothesized by Talbot et al.’s [52] study on genetic structure in rabid and non-rabid raccoons.

As noted by Nadin-Davis et al.’s [12] study, red fox populations may have limited potential to maintain the ARVV on the long term, but may contribute to rabies viral persistence in areas of red and Arctic fox sympatry. They may also contribute in spreading the rabies virus along certain geographical corridors, one of which being the northern portion of Quebec and Quebec-Labrador in Canada. This region has been implicated in a number of southerly rabies propagation waves [16–18]. The findings from our study, showing low genetic differentiation across the Quebec-Labrador Peninsula, further support the hypothesis of red foxes constituting a bridge for the large-scale propagation of rabies from the Arctic enzootic regions to southern areas. A conceptual modelling approach in Alaska suggests a potential shift of ARVV transmission from Arctic foxes to red foxes following an increase of the interactions between the two species [10, 19]. Southward rabies propagation through red foxes could therefore become more common in the future. Given the lack of resistance to movement observed in red fox and the ARVV southward propagation potential, early detection of ARVV outside enzootic areas become important to efficiently manage the risks. However, a large portion of the northern Canada is largely inaccessible for surveillance and management efforts. In this context, enhanced vigilance, or surveillance efforts of ARVV in red foxes near large population centers in southern Canada may become of increasing importance.

## Supporting information

S1 Table Information about red fox (*Vulpes vulpes*) specimens used in this study. Genotypes at 15 microsatellite markers, geographic coordinates rounded to the second decimal, year of collection, species and sex for all specimens used in this study. Missing data are labelled “NA”.
(XLSX)

S1 Appendix Outputs from the CLUMPAK software [33] and the ‘spca’ function from the ‘adegenet’ package [35] in R 4.2.1. Graphs showing likelihood and Delta K values for each value of number of genetic clusters (K) for red fox samples from Sites 1–4, for all red fox samples, for females red fox samples and for male red fox samples (Outputs 1–10), and graphs showing various results from the spatial principal components analyses for all red fox samples, for females red fox samples and for male red fox samples (Outputs 11–16).
(DOCX)
