# Supplementary material for: Low levels of genetic differentiation and structure in red fox populations in Eastern Canada and implications for Arctic fox rabies propagation potential
Source: PLoS One. 2023 Jun 6;18(6):e0286784. doi: 10.1371/journal.pone.0286784 (PMC10243632; doi:10.1371/journal.pone.0286784)
Supplement: S1 Appendix — Graphs showing likelihood and Delta K values for each value of number of genetic clusters (K) for red fox samples from Sites 1–4, for all red fox samples, for females red fox samples and for male red fox samples (Outputs 1–10), and graphs showing various results from the spatial principal components analyses for all red fox samples, for females red fox samples and for male red fox samples (Outputs 11–16). (DOCX) [file pone.0286784.s002.docx]

**Output 1.** Likelihood for values of number of genetic clusters (K) for red fox samples collected in Site 1 (Fig. 1A).

**Output 2.** Likelihood for values of number of genetic clusters (K) for red fox samples collected in Site 2 (Fig. 1A).

**Output 3.** Likelihood for values of number of genetic clusters (K) for red fox samples collected in Site 3 (Fig. 1A).

**Output 4.** Likelihood for values of number of genetic clusters (K) for red fox samples collected in Site 4 (Fig. 1A).

**Output 5.** Likelihood for values of number of genetic clusters (K) for all red fox samples.

**Output 6.** Delta K for values of number of genetic clusters (K) for all red fox samples.

**Output 7.** Likelihood for values of number of genetic clusters (K) for female red fox samples.

**Output 8.** Delta K for values of number of genetic cluster (K) for female red fox samples.

**Output 9.** Likelihood for values of number of genetic clusters (K) for male red fox samples.

**Output 10.** Delta K for values of number of genetic clusters (K) for male red fox samples.

**Output 11.** Connection network, three different representations of map of scores and eigenvalues barplot from the spatial principal components analysis of all red fox samples (an error in the function prevented the eigenvalues decomposition plot from drawing).

**Output 12.** Global test statistic (black bar), and reference distribution (barplot) from the spatial principal components analysis of all red fox samples.

**Output 13.** Connection network, three different representations of map of scores, eigenvalues barplot and eigenvalues decomposition plot from the spatial principal components analysis of female red fox samples.

**Output 14.** Global test statistic (black bar), and reference distribution (barplot) from the spatial principal components analysis of female red fox samples.

**Output 15.** Connection network, three different representations of map of scores, eigenvalues barplot and eigenvalues decomposition plot from the spatial principal components analysis of male red fox samples.

**Output 16.** Global test statistic (black bar), and reference distribution (barplot) from the spatial principal components analysis of male red fox samples.
